# Editorial: Impact of viral co-infection on cellular or human health and its clinical outcome

**DOI:** 10.3389/fcimb.2024.1399184

**Published:** 2024-04-03

**Authors:** Debsopan Roy, Nilanjan Chakraborty

**Affiliations:** Virus Research Laboratory, ICMR-National Institute of Cholera and Enteric Diseases (NICED), Kolkata, West Bengal, India

**Keywords:** co-infection, virus, pathogenicity, immunomodulation, clinical investigation

Co-infection is a phenomenon that occurs when a host is infected by two or more pathogens, such as viruses, bacteria, fungi, and protozoa, either at the same time or sequentially. This phenomenon is widespread in nature and can lead to several complications, including altered viral pathogenicity, disrupted host defence, and mixed-up clinical symptoms. The presence of multiple pathogens not only makes disease diagnosis and treatment more complicated, but it can also pose a significant threat to public health, especially in cases where the pathogens can interact and amplify each other’s effects. When two viruses interact with each other, five possible patterns of interaction can be observed: interference, synergy, non-interference, dependence assistance, and host–parasite relationship. Among these, interference is the most common interaction observed in co-infection, where one virus competes with another to suppress its replication. When a virus is classified as non-interference, it means that it does not affect or interfere with other viruses that may be present in the same host. This is usually observed when viruses have different preferences for infecting particular tissues. In cases of viral infections in humans or animals, it is possible to encounter a non-disease-causing virus within the host, and their relationship with a disease-causing virus is considered to be distinct and separate. This Research Topic examines clinical outcomes and changes in the immune system caused by bacterial-viral co-infections. Focus is given to tuberculosis with HIV co-infection, as well as co-infections with SARS-CoV-2, human polyomavirus, influenza virus, and human T-cell leukaemia virus type 1. These studies may uncover new insights into viral pathogenesis.

Tuberculosis and HIV/AIDS are the preeminent infectious diseases afflicting countries with limited resources. Mycobacterium tuberculosis and HIV can worsen immunological dysfunction in individuals, potentially resulting in premature mortality if untreated as well as HIV infection may increase susceptibility to latent tuberculosis. The previous study by Takhar et al. (2018) individuals with AIDS exhibit a significant depletion of CD4+ T cells, not only in their blood but also in lymphoid tissues and mucosa. This characteristic is considered the primary manifestation of immunosuppression in such individuals. This depletion is a crucial factor contributing to the elevated risk of developing active tuberculosis. Tan et al., found that persistent HIV infection among TB-infected patients can lead to a progressive deterioration of the immune system, resulting in increased morbidity if left untreated. The vulnerability of HIV/TB co-infected patients is much more life-threatening in comparison with only HIV-infected patients. HIV/TB co-infection showed different functional profiles of cytokine-secreting cells compared to TB and HIV alone, despite lower cell numbers. This suggests distinct immune responses in co-infection. It was also observed that CD8+ T cells that secrete TNF-α can be a better indicator to evaluate early efficacy of anti-TB treatment in HIV/TB co-infected patients. So understanding the clinic-immunological alteration of HIV/TB-infected patients is the need of the hour.

In the past few years, we have experienced the devastating spread of SARS-CoV-2, which has impacted populations worldwide and resulted in significant illness and death. Influenza is another serious respiratory infection that affects people globally. Although both infections pose significant health risks, little is known about the clinical aspects of co-infection between influenza and COVID-19. The symptoms of co-infection are almost identical to those of people infected with either virus alone. Previous research of Kong et al. (2022) revealed that getting vaccinated against influenza may offer protection against co-infection with influenza and COVID-19. In this Research Topic, a case report by Liang et al., sheds light on the type specificity of the influenza virus in COVID-19 patients. A negative PCR test for influenza infection made it difficult to diagnose until next generation sequencing (NGS) was conducted on BALF samples, leading to a prompt and accurate diagnosis. This study highlights the potential value of NGS in the timely diagnosis of patients with long COVID and their mixed infections. The report focuses on a specific cohort and emphasizes clinical parameters to provide new insights.

A previous investigation of Olbei et al. (2021) showed that SARS-CoV-2 can directly lead to the reactivation of herpesviruses by interacting with herpesvirus elements or regulating host factors involved in reactivation-related cellular signalling pathways, which can then trigger a cytokine storm but the immunological mechanisms is still unclear. In a recent investigation of Lu et al., published in this Research Topic, a diverse range of herpesvirus infections were identified among individuals infected with SARS-CoV-2. This finding represents a significant milestone in clinical investigations aimed at advancing our understanding of this subject matter. Such insights are critical for advancing our knowledge of the complex interplay between these viral pathogens and their impact on human health and also serve as an important reference point for future research in this field.

Formerly classified under the family *Papovaviridae*,on account of their similar morphology and genome organization, *human papillomaviruses* (HPV) and *polyomaviruses* (HPyV) are currently assigned to the families *Papillomaviridae* and *Polyomaviridae*, respectively. Both viral families have been linked to the development of human cancers. While studies on the interaction between these two virus types are limited, previous research of Guidry et al (Guidry and Scott, 2017 ). indicates that co-infection by both may enhance the transforming properties of papillomavirus, regardless of whether they are present in the same or different locations. One article in this Research Topic by Laine et al., presents interesting insights into these viral co-infectivity that could change future research in viral pathogenesis. They represented that HPyV/HPV co infection is prominently visible in different tissue organization but neither any virus drastically impair any significant clinical outcome on the host.

*Human T lymphotropic virus* type 1 (HTLV-1) is a retrovirus that causes a lifelong T-cell infection in humans, which has a significant impact on the host immune response. This virus induces a wide range of clinical manifestations, including inflammatory conditions such as neuronal damage along with life-threatening leukaemia. HTLV-1-infected individuals are often co-infected with a variety of pathogens, and the virus itself may exacerbate the response of one arm of the immunological system. Recently, a meta-analysis by Schierhout et al. (2020) showed that HTLV-1 infection is linked to an increased risk of death from various life-threatening clinical conditions. These findings underscore the importance of continued research on HTLV-1 and the development of effective treatment strategies to improve outcomes for those who are infected. A paper in this Research Topic by Dugardin et al., reports for the first time on the association between HTLV-1 and dysthyroidism in a large sample and initiate the fundamental approaches of systematic exploration of thyroid function of patients with HTLV-1 to aid in the early detection and management of dysthyroidism.

It is clear that microbial interactions play a role in increasing the severity of infectious diseases and can lead to higher rates of illness and death. We are pleased to present this Research Topic dedicated to the topic of viral co-infection, which has been gaining attention in the field of infectious diseases. We hope you find it informative and interesting.

## Author contributions

DR: Writing – original draft, Writing – review & editing. NC: Writing – original draft, Writing – review & editing.
